# A genome-wide search replicates evidence of a quantitative trait locus for circulating angiotensin I-converting enzyme (ACE) unlinked to the ACE gene

**DOI:** 10.1186/1755-8794-1-23

**Published:** 2008-06-10

**Authors:** Colin A McKenzie, Xiaofeng Zhu, Terrence E Forrester, Amy Luke, Adebowale A Adeyemo, Nourdine Bouzekri, Richard S Cooper

**Affiliations:** 1 Tropical Metabolism Research Unit, University of the West Indies, Kingston, Jamaica; 2 Department of Preventive Medicine and Epidemiology, Loyola University, Chicago, USA; 3 Department of Paediatrics/Institute for Child Health, University of Ibadan, Ibadan, Nigeria; 4 Institute of Biological Anthropology, University of Oxford, Oxford, UK

## Abstract

**Background:**

Angiotensin I-converting enzyme (ACE) plays an important role in cardiovascular homeostasis. There is evidence from different ethnic groups that circulating ACE levels are influenced by a quantitative trait locus (QTL) at the ACE gene on chromosome 17. The finding of significant residual familial correlations in different ethnic groups, after accounting for this QTL, and the finding of support for linkage to a locus on chromosome 4 in Mexican-American families strongly suggest that there may well be QTLs for ACE unlinked to the ACE gene.

**Methods:**

A genome-wide panel of microsatellite markers, and a panel of biallelic polymorphisms in the ACE gene were typed in Nigerian families. Single locus models with fixed parameters were used to test for linkage to circulating ACE with and without adjustment for the effects of the ACE gene polymorphisms.

**Results:**

Strong evidence was found for D17S2193 (Z_max _= 3.5); other nearby markers on chromosome 17 also showed modest support. After adjustment for the effects of the ACE gene locus, evidence of "suggestive linkage" to circulating ACE was found for D4S1629 (Z_max _= 2.2); this marker is very close to a locus previously shown to be linked to circulating ACE levels in Mexican-American families.

**Conclusion:**

In this report we have provided further support for the notion that there are QTLs for ACE unlinked to the ACE gene; our findings for chromosome 4, which appear to replicate the findings of a previous independent study, should be considered strong grounds for a more detailed examination of this region in the search for genes/variants which influence ACE levels.

The poor yields, thus far, in defining the genetic determinants of hypertension risk suggest a need to look beyond simple relationships between genotypes and the ultimate phenotype. In addition to incorporating information on important environmental exposures, a better understanding of the factors which influence the building blocks of the blood pressure homeostatic network is also required. Detailed studies of the genetic determinants of ACE, an important component of the renin-angiotensin system, have the potential to contribute to this strategic objective.

## Background

Angiotensin I-converting enzyme (ACE) plays an important role in the maintenance of cardiovascular homeostasis [1-3]. Complete and tissue-specific ACE knockout mouse models have demonstrated the important roles of both circulating and endothelial ACE, and of interactions between ACE activity and dietary salt intake in the determination of blood pressure [4,5]. There are also complementary data which suggest that responses to thiazide diuretics may be related to genetically-determined variation in ACE activity [6,7]. Clinical trials have shown that the use of ACE inhibitor medication in cardiovascular and renal disease, and among persons at high risk for heart disease confers benefits that may be independent of blood pressure lowering [8-14]. More recently, it has been suggested that the use of ACE inhibitor medications is associated with lowered risk of developing Type 2 diabetes mellitus [15]. The apparent difficulty that has been experienced thus far in identifying susceptibility genes for essential hypertension [16], and the possibility that genetically-determined variation in ACE activity might influence either risk of disease, or of outcomes among patients treated with ACE inhibitor or other medications, continues to fuel interest in defining the mechanisms that influence ACE activity.

ACE is a membrane-bound Zn^2+ ^metallopeptidase; the circulating form is derived from tissues by cleavage of the C-terminal transmembrane stalk [17]. ACE levels in plasma display major gene determination and there is evidence that there is a quantitative trait locus (QTL) at or near the ACE gene on chromosome 17 [18-21]. The availability of extensive data on polymorphic sites within the gene has facilitated the identification, in Caucasians, of intervals, defined by recombination breakpoints that are likely to contain variants which influence circulating ACE levels [22-24]. Similar analyses of ACE polymorphisms in Nigerian families [25] suggest that multiple variants influence ACE levels; the major effect appears to be contained within an intragenic region previously identified among white Europeans, with an additional, minor effect localised to 5' non-coding sequence. The mechanism by which these variants influence ACE levels remains unknown. For instance, the variants that have been identified in the critical regions would not be expected to affect the expression of the gene or the rate of cleavage of the C-terminal stalk [24,25]; a possibility is that variants in other genomic regions might be involved in the determination of ACE levels.

In addition to effects localised to the ACE gene locus, there is evidence which suggests that there are other loci, unlinked to the ACE gene, which influence circulating ACE levels. In analyses of black Jamaican families [18], it was estimated that the ACE gene-linked QTL accounted for 27% of the total variability while an unlinked QTL accounted for 52% of the variability. In British Caucasian families [22] it was estimated that the ACE gene-linked QTL accounted for 36% of the total phenotypic variance while residual familial correlations accounted for 10% of the variance. In analyses of both French Caucasian [24] and Nigerian families [25] significant residual familial correlations have also been found (representing 10.5% and 19% of the phenotypic variance respectively) after accounting for the ACE-linked QTL. More recently, in studies of Mexican-American families [26], a putative QTL for ACE on chromosome 4 has been identified. These results from several different ethnic groups provide support for the notion that loci unlinked to the ACE gene may influence circulating ACE.

Given that there is strong evidence of genetic determination of circulating ACE levels we were interested in using model-based analysis to explore whether we could identify a QTL for ACE levels on chromosome 17 and, if that were possible, to use the same approach to explore whether we could identify additional QTLs for ACE levels unlinked to chromosome 17. In an effort to do this we have conducted an autosomal genome-wide search for loci linked to plasma ACE levels in a dataset comprising 2,079 members of 289 Nigerian families. Two-point linkage analysis under a model with fixed parameters was used to evaluate support for linkage to plasma ACE levels with and without adjustment for the effects of the ACE-linked QTL.

## Methods

### Participant Recruitment, Survey Methods, and ACE measurements

The recruitment, phenotyping, and measurement of ACE levels in these families have been described in detail previously. Briefly, the sampling frame for this study was provided by the International Collaborative Study on Hypertension in Blacks (ICSHIB) [27,28]. Nuclear families were identified through a middle-aged proband and his/her spouse; all available first-degree relatives were enrolled as were available half-sibs in both the proband and offspring generations [29]. The medical history and family pedigree were obtained and heights, weights, and blood pressures were measured according to a standardized protocol [27,28]. Participants with hypertension were offered treatment after detection at the screening exam. ACE concentration was determined using a previously published sandwich ELISA [30] with minor modifications.

The protocol was approved by the IRB at Loyola University and the Ethics Committee, University College Hospital, Ibadan. Informed consent was presented in Yoruba or English and was obtained from participants by local staff.

### Genotyping

DNA was extracted from buffy coats and submitted to the NHLBI Mammalian Genotyping Service, Marshfield, WI. Tandem repeat markers from Marshfield "Set 10" [31], with an average map distance of 10 cM, were typed. There were 378 autosomal markers available for the analyses reported here; some of these data have been used previously in a linkage analysis of blood pressure [32]. Allele frequencies were estimated from the data using a counting algorithm implemented in recode [33].

High-resolution mapping of a putative ACE QTL linked to the ACE gene has been carried out in a subset of families recruited as part of the ICSHIB project. Briefly, genotypes were determined for 35 biallelic markers in or near to the ACE gene. Twenty-two markers were typed by DNA re-sequencing [25]; the remainder were typed by PCR/RFLP analysis [22,34].

### Statistical Analysis

Serum ACE level was treated as a continuous trait since none of the participants were receiving consistent antihypertensive treatment at the baseline exam. Linear regression was used to adjust ACE level for age and sex (Quantitative Trait 1, QT1). In the subset of families who had genotype data for the ACE gene markers described above, linear regression was used to adjust ACE level for age, sex, and for the effects of the ACE gene-linked QTL (Quantitative Trait 2, QT2); biallelic ACE gene markers[25] were selected for inclusion in the linear regression model, without explicit specification of linkage disequilibrium, using a manual backward stepwise procedure. Four markers (C7715T, A23495G, 29349delT, A31958G) were included in the final model. QT1 and QT2 were separately standardised to have a mean of zero and a variance of one prior to linkage analysis.

Two-point linkage analyses for QTLs influencing either QT1 or QT2 were conducted under a fixed major gene model which assumed a two-allele QTL (alleles A and a), genotype-specific means, *μ*_AA _= – 1.9, *μ*_Aa _= 0.5, and *μ*_aa _= 2.5, frequency of allele A (associated with lower trait values) = 0.75, and a common within-genotype variance, σ^2 ^= 0.5. A model with widely spaced genotype means, and a relatively low allele frequency corresponds to a high penetrance ratio between genetic and non-genetic cases for discrete traits [35]. The model parameters were selected based on trials conducted on a previously-reported set of Jamaican families [18]. Likelihood computations were performed using the mlink routine of the programme fastlink [36-40].

We used merlin [41] to simulate 20 replicates of each autosome under the null hypothesis of no linkage using the actual pedigree structures and marker data from the Nigerian family dataset. The replicate datasets were then analysed using mlink. This procedure allows us to develop a very preliminary, indicative estimate of genome-wide false positive rates at different maximum lod score thresholds given the pedigree structures, patterns of missing information, allele frequencies, and marker locations actually present in our dataset. File formatting for this exercise was carried out using mega2 [42].

## Results

### Characteristics of the study sample

The dataset available for this analysis included 2,079 persons in 289 pedigrees. The median pedigree size was six (interquartile range, IQR 5 – 8), the median number of sibships of at least size two per pedigree was one (IQR 1 – 2), and the median number of siblings per sibship was four (IQR 2 – 6). The analysis of QT1 was conducted on families with ACE levels that were also typed for the Marshfield markers. The analysis of QT2 was conducted on a subset of these families that were also typed for ACE gene markers. The characteristics of the QT1 sample and of the QT2 subset are shown in Table 1. A full listing of the numbers and types of relative-pairs is available [see Additional file 1]. The subgroup with ACE markers typed has a greater proportion of men (P < 0.05), is older (P < 0.0001), slightly heavier (P < 0.01), and has higher blood pressures (P < 0.0001 for both systolic and diastolic blood pressure) when compared to the overall study sample. There was, however, no significant difference in ACE concentration between the subgroup and the overall family sample.

**Table 1:** Mean ± SD values for age, blood pressure, anthropometric measures, and ACE levels

	All family members	ACE markers typed
N (% male)	2063 (48.7)	544 (54.4)
Age (years)	36.8 ± 19.4 _(n = 2060)_	44.1 ± 18.7 _(n = 544)_
BMI (kg/m^2^)	21.0 ± 4.6 _(n = 1712)_	21.7 ± 4.5 _(n = 529)_
WHR^a^	0.85 ± 0.057 _(n = 552)_	0.88 ± 0.067 _(n = 118_)
Systolic Blood Pressure (mm Hg)	120.9 ± 26.8 _(n = 1700)_	131.2 ± 27.6 _(n = 543)_
Diastolic Blood Pressure (mm Hg)	74.0 ± 16.9 _(n = 1700)_	79.0 ± 17.8 _(n = 543)_
ACE (ng/ml)	604.7 ± 213.2 _(n = 1169)_	612.7 ± 213.0_(n = 544)_

^a ^WHR – waist circumference:hip circumference ratio

### Linkage evidence

**Figure 1:**
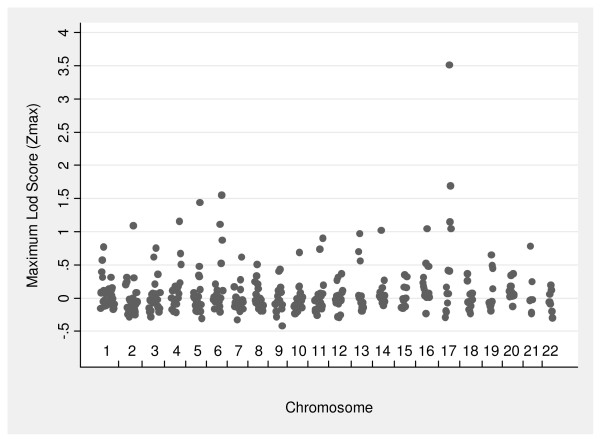
Maximum lod score (Z_max_) values by chromosome for QT1 (ACE levels adjusted for age and sex).

Figure 1 shows the maximum lod score (Z_max_) by chromosome for each of the autosomal markers tested for linkage to QT1. The strongest evidence for linkage was found on chromosome 17 where one marker (D17S2193) achieved a Z_max _value of 3.51 and three other markers had Z_max _values greater than one including one marker (D17S2195) with a Z_max _value greater than 1.5 (Table 2). These markers lie approximately 4.1 and 13.5 Mb away from the ACE gene locus which we have previously shown to be linked to circulating ACE levels in different ethnic groups [18,43]. Only one other marker (D6S1277) attained a Z_max _value greater than 1.5. No other markers on chromosome 6 had Z_max _values greater than one.

**Table 2:** Loci with Z_max _values > 1.0 for QT1 (plasma ACE adjusted for age and sex)

**Chr**.	**Marker**	**Z_max_**	**Theta**
2	D2S1353	1.09	0.2
4	D4S2431	1.16	0.2
5	D5S820	1.44	0.2
6	GATA184A08	1.11	0.2
	D6S1277	1.55	0.2
14	D14S599	1.02	0.2
16	GATA138C05	1.04	0.2
17	D17S2193	3.51	0.1
	D17S1301	1.15	0.2
	D17S2195	1.69	0.2
	D17S784	1.04	0.2

**Figure 2:**
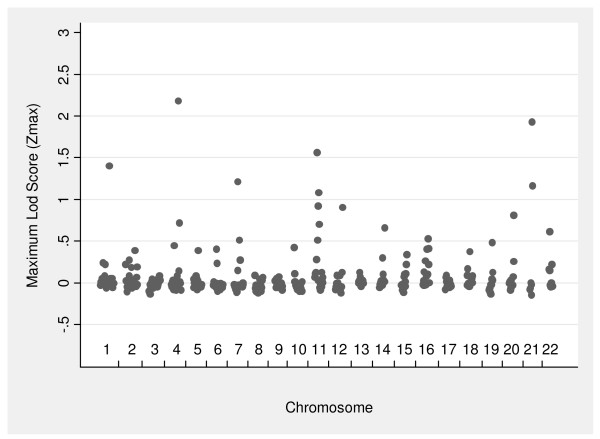
Maximum lod score (Z_max_) values by chromosome for QT2 (plasma ACE levels adjusted for age, sex, and ACE gene markers).

The Z_max _values by chromosome for the 2-point linkage analyses of QT2 are shown in Figure 2. For this trait, seven markers on five chromosomes achieved Z_max _values greater than one. The strongest evidence for linkage to QT2 is found on chromosome 4 where D4S1629 had a Z_max _value of 2.18 (Table 3). Chromosome 11 and chromosome 21 each had one marker (of two markers with Z_max _values greater than one) with Z_max _values greater than 1.5 (Table 3). Chromosome 4 is the only chromosome on which there is a Z_max _value > 1.0 for both QT1 (D4S2431, Z_max _= 1.16) and QT2 (D4S1629, Z_max _= 2.18).

**Table 3:** Loci with Z_max _values > 1.0 for QT2 (plasma ACE levels adjusted for age, sex, and the effects of the ACE gene locus)

**Chr**.	**Marker**	**Z_max_**	**Theta**
1	D1S1589	1.40	0.01
4	D4S1629	2.18	0.0
7	D7S1818	1.21	0.01
11	D11S1392	1.56	0.0
	D11S4459	1.08	0.05
21	D21S1411	1.93	0.0
	D21S1446	1.16	0.01

### Preliminary simulation-based estimates of false positive rates

We conducted a limited simulation exercise in order to make a preliminary, indicative estimate of the genome-wide false positive rates associated with various Z_max _thresholds observed in our study. For the QT2 dataset (i.e. the subgroup typed for both genome-wide microsatellite markers as well as ACE gene SNPs) we found that Z_max _values of 1.0, 1.5, and 2.0 would be expected to be associated with 5.8, 1.5, and 0.2 false positives per genome screen. A Z_max _threshold of 1.6, for this dataset, represents 'suggestive linkage' [44]. That is, this threshold is associated with 1 false positive or less per genome screen (95% confidence interval, CI 0.56 – 1.43). A more conservative threshold would be a Z_max _of 1.75 (0.6 false positives per genome screen, 95% CI = 0.26 – 0.94). Using this empirically-derived set of thresholds for QT2, we then have at least 2 markers (D4S1629 and D21S1411) which show 'suggestive linkage', and one marker (D11S1392) which just fails to meet that standard. Furthermore, it should be noted that the Z_max _value of 2.18 for D4S1629 is associated with a false positive rate of 0.1 (95% CI 0.00 – 0.24) per genome screen which is an order of magnitude beyond the threshold for suggestive linkage and even begins to approach the false positive rate for 'significant linkage' (i.e. 0.05 false positives per genome screen).

## Discussion

There is strong evidence of genetic determination of circulating ACE and there is also evidence that this genetic determination may include loci unlinked to the ACE gene on chromosome 17. After adjustment for the effect of the ACE gene locus we found that there was modest support for linkage between circulating ACE levels and microsatellite markers on chromosomes 4, 11, and 21. D4S1629 had a Z_max _value of 2.18 at which we estimated the genome-wide false positive rate to be consistent with "suggestive linkage"; as previously proposed [44]. The markers on chromosomes 11 and 21 had somewhat lower Z_max _values but in each case there was another marker nearby with Z_max _> 1.0.

In our analysis we adjusted circulating ACE levels for SNPs in the ACE gene (which accounted for approximately 26% of the total variation in ACE levels) and then performed linkage analysis using fixed effects maximum likelihood (FEML); adjustment for known sources of familial correlations is known to improve power for detection of QTLs [45-49]. We did not, however, observe any marker with Z_max _> 3.0 for the adjusted trait (i.e. QT2); this is unsurprising and is likely to be due to the relatively small number of families typed for both microsatellite markers **and** ACE gene SNPs. In order to examine the power of the QT2 dataset, we have performed simulations using slink [50,51]; for a moderately informative marker (heterozygosity 0.76, 1.7% missing data) tightly linked (θ = 0.001) to QT2, our pedigrees would generate Z_max _≥ 2.0 and ≥ 2.5 in only 46% and 29% of 1000 replicates respectively. It is also possible that the use of a single, possibly mis-specified, model may have had an impact on the evidence for linkage; the use of model-free or non-parametric methods is motivated primarily by this concern [52,53]. Nevertheless, parametric or model-based methods can be powerful for detection of linkage even though estimates of the recombination fraction may be biased upwards [35,54-58]. In this analysis we had the benefit of a "positive control" in the form of the previously-described QTL for circulating ACE within or near to the ACE gene. Our finding of significant linkage (Z_max _~ 3.5) between microsatellite markers near the ACE gene and circulating ACE levels, the first such report using FEML with microsatellites from a sparse map, suggests that models with widely-spaced genotype means, and a relatively low allele frequency (corresponding to high penetrance ratios between genetic and non-genetic cases for discrete traits) should not lead to incorrect inferences regarding detection of linkage signals for circulating ACE. With all of these considerations in mind, it seems a reasonable proposition that our finding of "suggestive linkage" to D4S1629 represents a replication of the identification of a putative QTL for ACE on chromosome 4 in Mexican-American families [26]. By itself the lod score of 2.18 for D4S1629 in our study represents considerable *prima facie *evidence of linkage even under the conservative proposals, for multifactorial disease, that have previously been made [44]; when one considers that D4S1629 is only 6.2 Mb away from D4S1548 which was the marker found to be significantly linked to ACE levels among Mexican-American families the evidence appears to be compelling. Nevertheless, further analyses of other datasets, including meta-analyses, will be required in order to demonstrate the existence of an ACE QTL in this region beyond all reasonable doubt; it remains unclear what thresholds ought to be used in declaring "suggestive" or "significant" linkage for traits where the model is not well known. ACE QTLs unlinked to the ACE gene might influence ACE levels by affecting any one (or more) of a number of different processes (e.g. transcription, translation, post-translational modification); investigation of these mechanisms must, however, await fine mapping and identification of variants.

Although there is a considerable amount of information available about the physiology of blood pressure regulation, the search for genetic variants which influence risk of high blood pressure in the general population has, on the whole, been disappointing. This has been true even where investigators have moved away from classical family-based designs; in a recent large-scale genome-wide association study [59], not only were there no SNPs which showed strong association with hypertension, there were no replications of putative associations identified in previous studies. Doubtless, the nature of the blood pressure control network, with its many components, regulated feedback loops, and interactions with numerous environmental factors, contributes to this difficulty. It is possible that the variants being sought might be relatively common but associated with only small-to-moderate increases in risk. Such variants, while difficult to identify, may have large associated population attributable risks and thus remain important targets in efforts to define risk factors for common disease; studies may need to be of even larger size if greater within-sample homogeneity cannot be achieved. The importance of ACE and the renin-angiotensin system in long-term blood pressure regulation suggests that a better definition of the genetic determinants of activity in these systems may be helpful in improving our understanding of the processes which ultimately lead to high blood pressure. This is especially so since our ability to investigate and quantify the effects of well-known lifetime risk factor exposures (eg salt intake) is, by the nature of the exposure, severely limited.

## Conclusion

In this report we have shown that there is strong evidence that there may be loci, unlinked to the ACE gene, which influence circulating ACE levels. One of the QTLs identified in this analysis of Nigerian families appears to be the same as a QTL identified previously in Mexican-American families. Given the importance of the renin-angiotensin system in long-term blood pressure regulation, further characterisation of the genetic determinants of circulating ACE has the potential to improve our understanding of the mechanisms underlying the development of high blood pressure.

## Competing interests

The authors declare that they have no competing interests.

## Authors' contributions

This report is a sub-study within the International Collaborative Study of Hypertension in Blacks (ICSHIB). CAM conceived and designed the sub-study, analysed the data and drafted the manuscript. XZ advised on the statistical analyses. TEF, AL, and RSC were involved in the conception, design, and supervision of the parent study. AAA was involved in the design of, and recruited participants for the study. NB conducted the genotyping. All authors read and approved the final manuscript.

## Pre-publication history

The pre-publication history for this paper can be accessed here:



## Supplementary Material

Additional file 1 Relative pairs. A listing of the numbers of the different types of relative pairs found in the QT1 ("All family members") and QT2 ("ACE markers typed") datasets. Click here for file
