# Supplementary material for: A genome-wide search replicates evidence of a quantitative trait locus for circulating angiotensin I-converting enzyme (ACE) unlinked to the ACE gene
Source: BMC Med Genomics. 2008 Jun 10;1:23. doi: 10.1186/1755-8794-1-23 (PMC2442613; doi:10.1186/1755-8794-1-23)
Supplement: Additional file 1 — Relative pairs. A listing of the numbers of the different types of relative pairs found in the QT1 ("All family members") and QT2 ("ACE markers typed") datasets. [file 1755-8794-1-23-S1.doc]

Listing of numbers of relative pairs in the QT1 (“All family members”) and QT2 (ACE markers typed) datasets. The listing was generated using the software package SOLAR[[1]](#footnote-2)

|  |  |  |
| --- | --- | --- |
| Relationship | All family members | ACE markers typed |
|  |  |  |
|  |  |  |
| Unrelated | 655 | 141 |
| Self | 1144 | 544 |
| Parent-offspring | 879 | 214 |
| Siblings | 424 | 100 |
| Grandparent-grandhchild | 68 | 12 |
| Avuncular | 216 | 46 |
| Half-siblings | 291 | 61 |
| Grand avuncular | 4 | 1 |
| Half avuncular | 141 | 28 |
| 1^st^ cousins | 43 | 3 |
| Half grand avuncular | 3 | 1 |
| 1^st^ cousins, 1 rem | 11 | 1 |
| Half 1^st^ cousins | 26 | – |
| Half 1^st^ cousins, 1 rem | 2 | – |
|  |  |  |

1. Almasy L, Blangero J (1998) Multipoint quantitative trait linkage analysis in general pedigrees. *Am J Hum Genet* 62:1198-1211 [↑](#footnote-ref-2)
